# Porous carbons prepared from a novel hard wood composite waste for effective adsorption of Pb(ii) and Cd(ii) ions

**DOI:** 10.1039/d3ra06244a

**Published:** 2023-11-30

**Authors:** Shaimaa T. El-Wakeel, Nady A. Fathy, Magda E. Tawfik

**Affiliations:** a Water Pollution Research Department, National Research Centre 33 El Buhouth St, Dokki 12622 Giza Egypt shaimaa_tw@yahoo.com Shaimaa.tw@gmail.com; b Physical Chemistry Department, National Research Centre 33 El Buhouth St, Dokki 12622 Giza Egypt fathyna.77@hotmail.com; c Polymers and Pigments Department, National Research Centre 33 El Buhouth St, Dokki 12622 Giza Egypt magdaemileta@yahoo.com

## Abstract

In our previous investigations, a hard wood composite (HWC) was formulated by adding rice straw, as a filler to the recycled polystyrene foam waste at mass ratio (50/50) at 170 °C and pressed under 40 kPa. Here, the disposed HWC product as a model scrap was applied for production of porous carbons enclosed with graphene sheets. To attain this approach, HWC was hydrothermally carbonized (S1) followed by either post-heat treatment (S2) or potassium hydroxide (KOH, S3) activation at 750 °C for 2 hours. The properties of prepared samples were evaluated using SEM, ATR-IR, and porosity measurements. The adsorption performance of the obtained porous carbons toward removal of lead (Pb(ii)) and cadmium (Cd(ii)) ions from aqueous solutions was investigated under different operating conditions like contact time, initial pH, initial metal ions concentration and adsorbent dose. Kinetic models such as pseudo-first order, pseudo-second order and intraparticle diffusion were used to analyze the adsorption data. Langmuir, Freundlich, Dubinin–Radushkevich and Redlich–Peterson isotherms were applied. Thermodynamics and regeneration studies were performed. The sample (S3) comprised a micro-mesoporous carbon structure encompassed by graphene sheets, with the largest total surface area (422 m^2^ g^−1^) and adsorption capacities for Pb(ii) and Cd(ii) ions of 207.9 and 119.6 mg g^−1^, respectively. The experimental adsorption data were best elucidated using Langmuir and pseudo second-order kinetic models. Thermodynamic experiments confirmed that adsorption is an endothermic and spontaneous process. Conclusively, the investigated HWC waste is a promising carbonaceous precursor for preparing effective porous graphene-carbons used in the removal heavy metals from their aqueous stream.

## Abstract 2

SEM figures describe conversion of a hard wood composite waste to porous carbons enclosed with graphene sheets.

## Graphical abstract

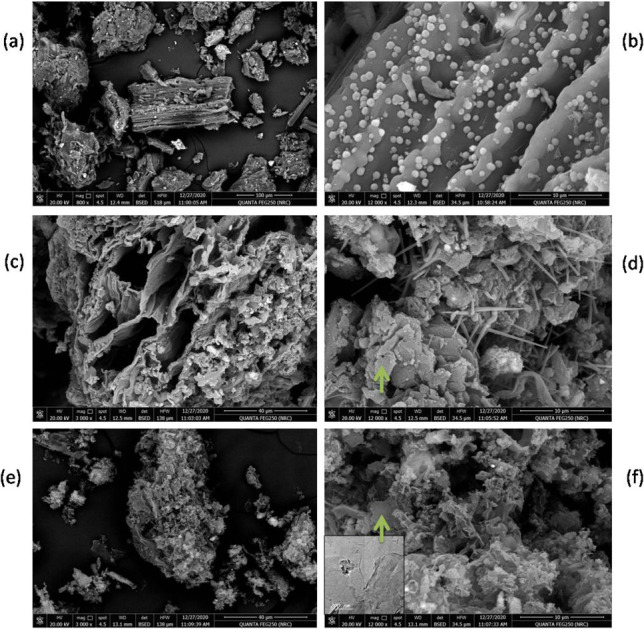

## 1. Introduction

Recently more toxic heavy metals have entered the environment, especially water bodies, due to the rapid development of industry where they are discharged as waste water from various industries including acid batteries, mining activities, electroplating and chemical industries.^1^ Heavy metals, besides their bioaccumulation, cannot be degraded in nature and can cause serious harmful effects when discharged to water systems and enter the food chain making a threat to human health.^2^ Toxic metals such as As(iii), Cd(ii) and Pb(ii) are known to be poisonous even at low concentrations and cause many diseases like autoimmune diseases and kidney damage leading to cancer and even death in severe cases.^3^ Both lead (Pb(ii)) and cadmium (Cd(ii)) are used extensively in numerous industrial applications and also they are well-known for their high toxicity.^4^ Lead ions are stated to cause many diseases in digestive and respiratory systems.^5^ Cadmium ions are more available as their compounds are more soluble than other heavy metals and their accumulation in the body causes bone and kidney diseases.^6^ Therefore, efficient techniques should be developed for lead and cadmium removal before their discharge into the environment. For this reason, different methods such as precipitation; ion exchange, electrochemical processes and adsorption have been adapted to heavy metal removal from water and waste water.^7^ Adsorption technology is favored because of low-cost, fast, simple operation and excellent performance.^8^

Adsorption of pollutants using carbon-based adsorbents has attracted a great attention due to their physicochemical features and porous structure.^7,8^ Accordingly, different adsorbents have been developed for removal of heavy metals such as porous carbon-based materials, including activated carbon/biochar, carbon nanotubes and graphenes analogues, which have been widely studied due to their wide distribution, large specific surface area, pore structure and low cost.^9–14^ Activated carbon, which can be obtained by carbonization and activation of very wide carbon rich precursors, is considered as a promising heavy metals adsorbent due to its relatively high mechanical strength.^15^

In recent years, the preparation of activated porous carbons from biomass feedstock through hydrothermal carbonization followed by chemical activation has received great attention^16,17^ and is considered as an alternative to thermochemical synthesis method to produce solid hydrochar materials with high concentrations of oxygenated functional groups at moderate temperatures (*ca.* 180–250 °C) under self-generated pressures at approximately 20–30 bars.^17^ Due to its operation at low temperature and being environmentally friendly, hydrothermal carbonization is more efficient than the conventional dry carbonization beside its performance in the enclosed system conditions. Post-carbonization also produces high porosity porous carbons which have better performance than commercial activated carbons in some instances.^16^

In recent years, production of porous carbons enclosed with graphene sheets or graphene-like carbon nanostructures is a hot and novel topic.^17–24^ Taking into consideration the conversion of a waste biomass into high value products, some attempts have already reported to design carbon-based nanostructured materials including graphene-like porous carbons from different biomass wastes by exploring ways to turn a biomass waste into a high value-product.^19–24^ Owing to their unique and tunable structural, mechanical, and chemical properties, these carbon materials paid much attention to be used as adsorbents in removing hazardous pollutants.^24^ These materials have also attracted a great deal of attention in various fields such as engineering, medical, material synthesis, electronics, energy and environment. In graphene, each carbon atom is attached to another carbon atom in the same plan through covalent bond and its single-sheets are connected by van der Waals forces. The presence of free π–π electrons and vacant reactive sites at the edges of aromatic rings gives remarkable characteristics of graphene allowing its potential for various applications such as bio-sensors, electronic devices, energy and purification.^19–25^

Besides the environmental gains, the hard wood composite (HWC) is emerged as an added value product. Owing to their fascinating properties like high mechanical, dimensional stability and sound resistance properties that qualified it to replace the natural wood in many daily applications, such as transportation, construction industries, military applications, building, packaging and furniture *etc.*^26^ HWC was formulated by adding rice straw waste, as filler, to the recycled polystyrene foam waste, as a matrix, (50/50 mass : mass), at 170 °C and pressing under 40 kPa.^26–29^ These studies discussed the possibility of using two of the most problematic wastes to formulate an added-value such as HWC. As well, HWC is a carbon-rich adsorbent which contains large quantities of various oxygenated functional groups in addition to the long life carbon matrix facilitating the sorption of target metal ions.^26,27^ At the end of its life span this hard wood composite lose its utilize and converted to a waste creating serious problems. Therefore, HWC is considered to be a recyclable waste, cheap and abundant on large scale. Avoiding the accumulation of this waste, the current study highlights the potential of utilizing HWC waste as a source material for synthesis of porous carbons enclosed with graphene sheets used in the removal some heavy metal pollutants from their aqueous stream. To our best knowledge, the use of HWC wastes to prepare porous carbons enclosed with graphene-like sheets to be used in removal of heavy metals from their aqueous stream is not yet studied.

In this work, three porous carbons-based adsorbents from the powder of HWC were prepared as the following recipe: (i) hydrochar was obtained from HWC *via* hydrothermal carbonization in presence of nickel and iron as catalysts at 150 °C for 2 h (S1), (ii) the obtained hydrochar was followed by thermally heated at 750 °C for 2 h (S2) and (iii) the obtained hydrochar was impregnated with KOH in mass ratio (1/4) and heated at 750 °C for 2 h (S3). The synthesized porous carbons were characterized by FE-SEM, ATR-IR and porosity determination. The adsorption performance of porous carbons obtained from HWC waste was evaluated by studying the adsorption of lead and cadmium ions as toxic metals through batch experiments at different operating conditions such as: pH, adsorbent dose and metals concentration. The adsorption rate and mechanism studies were demonstrated by applying various adsorption kinetics models.

## 2. Experimental

### 2.1. Materials

A hard wood composite waste (HWC) was obtained in laboratory.^26^ Lead and cadmium nitrate, nickel nitrate, ferrocene, potassium hydroxide were purchased from Sigma-Aldrich (Germany 99% purity). The working solutions of metal ions were prepared by appropriate dilutions of metal salt in de-ionized water immediately prior to their use.

### 2.2. Hard wood composite formulation

The HWC was prepared following the previous published works.^19–25^ For the HWC formulation, maleated polystyrene (PS) was prepared based on the recycled PS with maleic anhydride (MA) following the method previously published^19^ and used as a coupling agent. The recycled PS plus PS-*g*-MA were preheated in a Brabender Plasticoder at temperature 170 °C and at a rotor speed of 50 rpm. The dried ground rice straw (RS) was added gradually to the molten polymer combination (PS and PS-*g*-MA). The coupling agent (PS-*g*-MA) was added at the ratio 7.5% of the mass of PS used to provide better performance for the HWC.^17^ The constitutions of PS and PS-*g*-MA : RS at ratio (50/50 mass : mass) were kept constant throughout the whole study. For complete dispersion, the combination was mixed for 3–4 min to ensure a homogenous composite mixture. The obtained amorphous wood-composite liquor was removed from the Brabender, and then pressed into different thickness using laboratory hydraulic hot press at 170 °C and at pressure of 40 kPa for 10 min. Characterizations of HWC sample were studied in details in previous works.^19–25^

### 2.3. Preparation of porous carbon samples

HWC sample was crushed to powder in mesh size 0.1–0.5 mm. The powder waste as carbonaceous precursor was added to H_2_O/ethanol solution containing dissolved 2 wt% nickel nitrate and 2 wt% ferrocene. After stirring for 90 min at 80 °C, the mixture was put in a hydrothermal reactor of stainless steel (100 mL) and heated at 150 °C for 2 h then followed by filtration and drying at 80 °C overnight to give hydrochar sample S1. The obtained hydrochar sample was further treated thermally either with or without KOH at 750 °C under N_2_ gas in a tubular stainless steel reactor. The portion of hydrochar sample that carbonized directly at 750 °C for 2 h denoted as S2. The third portion of hydrochar was mixed firstly with KOH in mass ratio (1/4) before its carbonization at 750 °C for 2 h to produce S3.

### 2.4. Characterization of the prepared samples

The surface morphology of the obtained samples was determined using field-emission scanning electron microscope (FE-SEM, Quanta FEG-250). The morphology of graphene sheets in S3 was determined using high resolution-transmission electron microscope (HR-TEM, JEM-1230, Japan) operated at 120 kV. The most important surface functional groups of the prepared samples were determined by Fourier-Transform Infrared Spectroscopy with Attenuated Total Reflectance (FTIR-ATR), Brucker Vertex 80V with resolution 4 cm^−1^ in the range of 4000–400 cm^−1^. The textural parameters such as Brunauer–Emmett–Teller surface area (*S*_BET_, m^2^ g^−1^), total pore volume (*V*_P_, cm^3^ g^−1^) and average pore diameter (*r̄*, Å) were evaluated using nitrogen adsorption analysis at −196 °C BEL-Sorp, MicrotracBel Crop (Japan). Calculations of meso-and micropore volumes from BET measurements, where the total pore volume (*V*_P_) at *P*/*P*_o_ = 0.95 and micropore volume (*V*_micro_) computed at *P*/*P*_o_ = 0.3 and mesopore volume (*V*_meso_) = *V*_P_ − *V*_micro_. A pH meter (Hanna, HI 111) was utilized to measure the surface pH of solid sample which was conducted with hot water for 20 min. Metal ions concentrations were determined using inductively coupled plasma optical emission spectrometry (ICP-OES) (Agilent 5100).

### 2.5. Adsorption experiments

Predetermined mass of lead nitrate and cadmium nitrate salts were dissolved in distilled water to obtain Pb(ii) and Cd(ii) stock solutions each 1000 mg L^−1^. Different concentrations of the two metal solutions were prepared by diluting the stock solutions. Batch adsorption experiments were performed by adding a calculated mass of adsorbent to Pb(ii) or Cd(ii) solution with shaking (160 rpm) at room temperature (25 °C). At definite time intervals, samples of the solutions were collected and filtered with a filter membrane of a pore size of 0.45 μm. Then, samples were analyzed for Pb(ii) and Cd(ii) concentrations.

For the determination of metal ion loading after sorption, the amount of metal ion adsorbed at time *t* was calculated using1
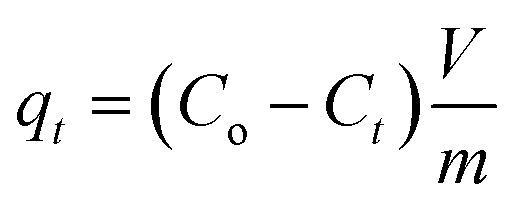


The removal efficiency and the adsorbed amount were calculated using the following equations:2
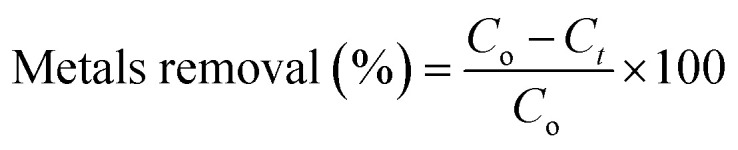
where *C*_*t*_ (mg L^−1^) is the metal ion concentration at time *t*, *q*_*t*_ (mg g^−1^) is the loading of the metal ion at time *t*, *C*_i_ is the initial metal ion concentration, *V* (mL) is the volume of the solution used and *m* (g) is the mass of the adsorbent used.

Several parameters were evaluated, including contact time (5–150 min), adsorbent dosage (0.05–3 g L^−1^), initial metals concentration (10–100 mg L^−1^) and initial pH value (pH = 2–6). All measurements were performed in triplicate and the mean value was calculated.

### 2.6. Adsorption kinetic and isotherm study

In this study, the solutions optimum pH was chosen to avoid the formation of metal ions precipitates. For kinetics experiment, the initial concentrations of Pb(ii) and Cd(ii) solutions were set to be 10 mg L^−1^ using the optimum adsorbent dose and after shaking at 160 rpm, samples were withdrawn at different time intervals.

To investigate the fit of adsorption isotherms, 50 mL metals solutions of different concentrations (25–300 mg L^−1^) were prepared and conducted with 100 mg adsorbent, while other adsorption conditions were the same as those of the kinetic experiments. The adsorbed solutions were analyzed for metal concentrations after filtration. The obtained data was modeled according to the pseudo-first-order (PFO), pseudo-second-order (PSO), and intraparticle diffusion (IPD) models, as expressed in coming eqn (3)–(5), respectively.

3 *q*_*t*_ = *q*_e_ − (1 − e^(−*K*_1_*t*)^)

4
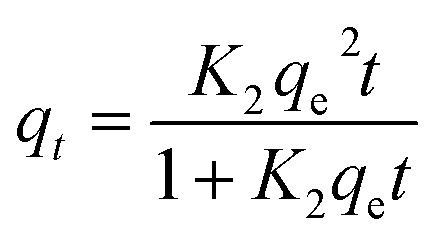

5 *q*_*t*_ = *k*_p_*t*^1/2^ + *C*

where, *q*_*t*_ is the amount of adsorbed metal (mg g^−1^) at equilibrium, *t* is the adsorption time (min), *K*_1_ (min^−1^), *K*_2_ (g mg^−1^ min^−1^) respectively, indicate the first and second order equilibrium constant, *k*_p_ is the intraparticle diffusion rate constant (mg g^−1^ min^−1/2^) and *C* is the intercept can be determined post plotting the related diagrams for each model.

The adsorption experimental data reached were modeled with Langmuir (eqn (6)), Freundlich (eqn (7)), Dubinin–Radushkevich (DR) (eqn (8)) and Redlich–Peterson (eqn (9)) isotherm models^3–5^ as expressed below:6
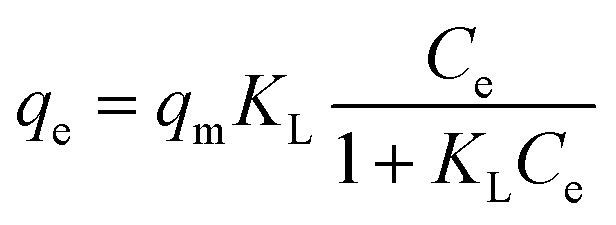

7 *q*_e_ = *K*_F_*C*_e_^1/*n*^

8 *q*_e_ = *q*_s_ e^(−*K*_DR_*ε*^2^)^

9
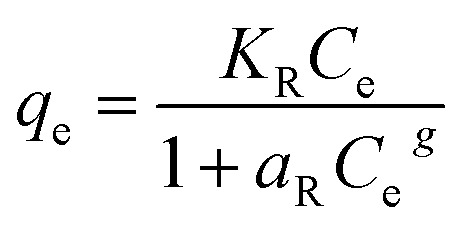
where *K*_L_ (L mg^−1^) is the Langmuir equilibrium constant related to the affinity of adsorption sites and *q*_m_ (mg g^−1^) represents the maximum theoretical monolayer adsorption capacity. *K*_F_ and *n* are the Freundlich adsorption constants which are related to the adsorption capacity and intensity, respectively. 1/*n* in the eqn (7) is a function of the strength of the adsorption process and the smallest of its value, the greatest is the expected heterogeneity. *K*_DR_ is a constant related to mean free energy (mol^2^ kJ^−2^), *q*_s_ (mg g^−1^) is a constant related to the adsorption capacity and *ε* representing Polanyi potential, which can be calculated from eqn (10):10
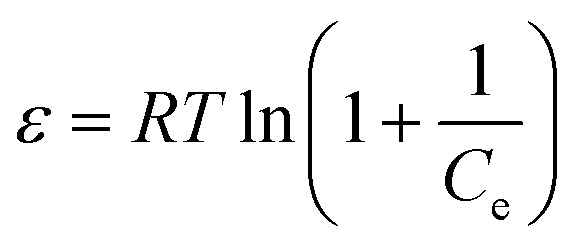
*K*_R_ (L mg^−1^) constant related to the adsorption capacity, *a*_R_ (L mg^−1^) constant related to the affinity of the binding sites and *g* exponent is related to the adsorption intensity which lies between 0 and 1.

The experimental data were fitted for non-linear kinetics and isotherm models using OriginPro 2016 Ver. 9.3.226. For the performance evaluation of the applied models, one of the most frequently calculated parameters is the coefficient of determination (*R*^2^) which was used to find the model of the best fit to the experimental data with values higher that are 0.96.

### 2.7. Adsorption thermodynamics study

The effect of different temperatures (313, 333 and 353 K) on Pb(ii) and Cd(ii) adsorption was studied and the experimental data obtained were applied to calculate the change of Gibbs free energy (Δ*G*^0^), the enthalpy change (Δ*H*^0^) and entropy change (Δ*S*^0^) according to Van't Hoff eqn (11)–(13):

11 Δ*G*^0^ = −*RT* ln *K*_d_

12 Δ*G*^0^ = Δ*H*^0^ − *T*Δ*S*^0^

13
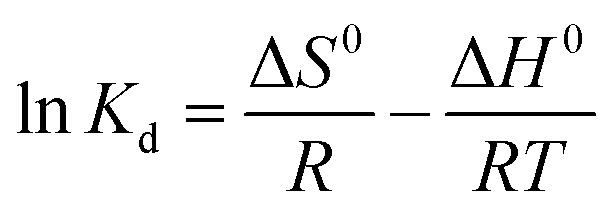
*K*_d_ is the distribution coefficient of the solute which is equal *q*_e_/*C*_e_, *R* is the universal gas constant (8.314 J mol^−1^ K^−1^), *T* (K) referred to the solution temperature.

### 2.8. Reusability and regeneration study

To test the adsorbent reusability for metals desorption, adsorption–desorption cycles of the prepared carbon samples were carried out. After adsorption of metal ions, the carbon sample-metal solution was filtered and metal concentration was determined. For desorption experiments, 10 mL of NaOH (1 M) was used as an eluent with 20 mg of carbon sample uptaken 10 mg L^−1^ of each Pb or Cd(ii) ions under shaking for 2 h. After that, the regenerated carbon sample was reused after being rinsed with deionized water and dried to constant weight. To evaluate the reusability of the adsorbents, the same sample was used for five successive recycles of adsorption/desorption.

## 3. Results and discussion

### 3.1. Characterization of the prepared samples

**Fig. 1:**
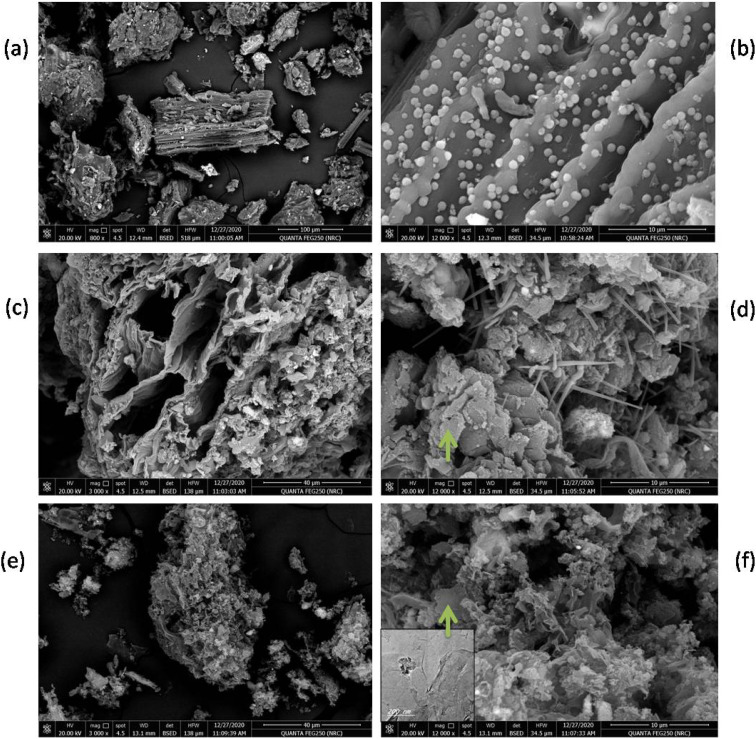
FE-SEM images of prepared samples (a & b) S1, (c & d) S2 and (e & f) S3 the with inset TEM image.

Field-emission scanning electron microscopy (FE-SEM) shows the morphology at low and high magnifications for hydrochar sample (S1), thermally treated hydrochar at 750 °C to give carbonaceous sample noted as (S2) and the activated with KOH at 750 °C to carbonaceous sample labeled as (S3) as seen in Fig. 1a–f. It can be seen clear differences in the morphologies due to the applied thermal treatment is varied. Surface of S1 is a smooth with rope-like of fibrous structure and contains spherical particles of the catalysts (Ni and Fe metals) and as shown in Fig. 1a and b. After direct thermal heating of S1 at 750 °C, the fibrous structure is opened in S1 forming a hollow fibrous structure and uniformly pointed noodles with outer diameters between 330 and 380 nm was obtained at S2 (Fig. 1c and d). Also, graphene-like layers were appeared as a result of Ni and Fe catalysts role which enhanced the graphitization process.^30^ During activation of S1 with KOH at 750 °C, the fibrous structure converted to graphene flakes (chips) over whole surface. Thus, loading of HWC waste with Fe and Ni catalysts was used to produce carbon hydrochar nanostructure with controlled morphology and functionality. Using catalysts with KOH at 750 °C also enhanced carbon hydrochar graphitization process through pyrolysis to form carbon enclosed with graphene-like sheets as reported previously.^18,19,23,31,32^

Moreover, a representative TEM investigation showed evidence for folding and wrinkling of graphene layers in S3 (see inset TEM in Fig. 1f) which is resemble to that appear also in S2, confirming the graphitization of S1 when exposed to direct heat treatment or KOH activation.

Adsorption of N_2_ gas is used to measure the evolved total porosity of the prepared samples. This analysis indicated that the graphene-like porous carbon sample (S3) has well-developed porosity as evident by their higher values of both total surface area and total pore volume as well as narrower mesopores as detected by mean pore diameter (*r̄*, nm) (Table 1). This affirms that porous graphene-like sheets are obtained after KOH activation. In addition, micro-and mesopore volumes data are calculated showing that S3 adsorbent sample own the largest values. Obviously the developed microporosity (*V*_micro_/*V*_P_ × 100 = 56.8%) and mesoporosity (*V*_meso_/*V*_P_ × 100 = 43.2%) are very high in S3. Hence, employing KOH as an activating agent after hydrothermal treatment of HWC can create micro–mesoporous structure as well as a catalyst for increasing the graphitization action. This result is in agreement with other reports.^18,23^ Accordingly, this sample exhibited considerably the highest adsorption capacity toward removal of heavy metals under investigation.

**Table:** pH values and total porosity of the prepared samples

Samples	pH_slurry_	*S*_BET_ a (m^2^ g^−1^)	*V*_P_ (cm^3^ g^−1^)	*V*_micro_ (cm^3^ g^−1^)	*V*_meso_ (cm^3^ g^−1^)	*r̄* (nm)
S1	4.2	8.5	0.011	0.002	0.009	5.22
S2	5.8	25.0	0.071	0.031	0.040	11.7
S3	8.5	422	0.394	0.224	0.170	3.73

a Brunauer–Emmett–Teller surface area (*S*_BET_), total pore volume (*V*_P_), micropore volume (*V*_micro_), mesopore volume (*V*_meso_) and pore diameter (*r̄*).

**Fig. 2:**
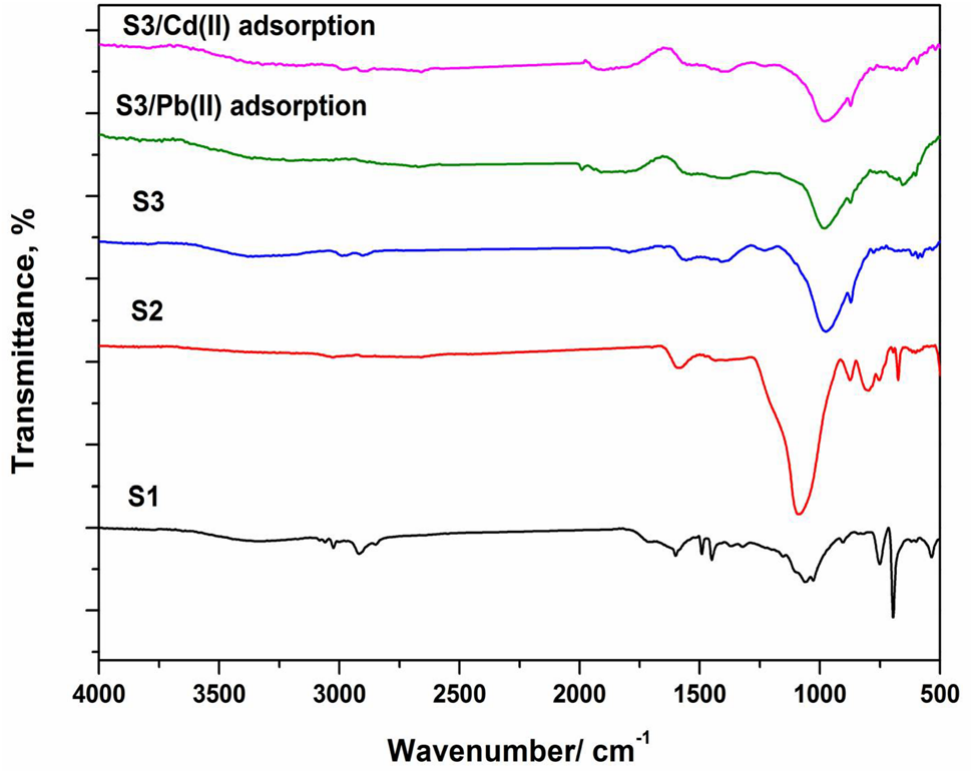
FTIR-ATR spectra of the obtained carbonaceous samples.

Surface pH (pH_slurry_) of the prepared samples was measured (Table 1) and found to be acidic for S1 and S2 while basic nature for S3. This confirms that negative charges surrounded S3 and hence strong electrostatic interactions with Pb and Cd ions could be happened significantly. However, FTIR-ATR analysis is applied to follow the change in the surface functional groups containing the main carbon and oxygen atoms as shown in (Fig. 2).^19,23,25,32^ Moreover, FTIR spectrum was recorded after adsorption of Pb and Cd(ii) ions on S3. Obviously, the direct thermal heating caused obvious changes where absorption bands of O–H at 3400 cm^−1^ are often disappeared whereas that of O–H at 1570 cm^−1^ is decreased slightly after KOH activation. The absorption band intensity of C–O in ethers, alcohols and aldehydes is increased and shifted to higher wavenumber between 950 and 1100 cm^−1^ for S2 and S3. Such band is ascribed to formation of epoxy and alkoxy groups stretching over graphene sheets. It is noticed that KOH activating agent led to a harsh effect on the surface functional groups located between 2000 and 500 cm^−1^ with preserving absorption band of O–H group at 1570 cm^−1^. After adsorption of targeted metal ions on S3, the oxygen functional groups related mainly to O–H and C–O groups (as Lewis base) were shifted remarkably to higher wavenumbers and reduced to lower intensities, indicating the adsorption of both metals *via* surface adsorption onto S3 (Fig. 2).

### 3.2. Adsorption performance of porous carbons

**Fig. 3:**
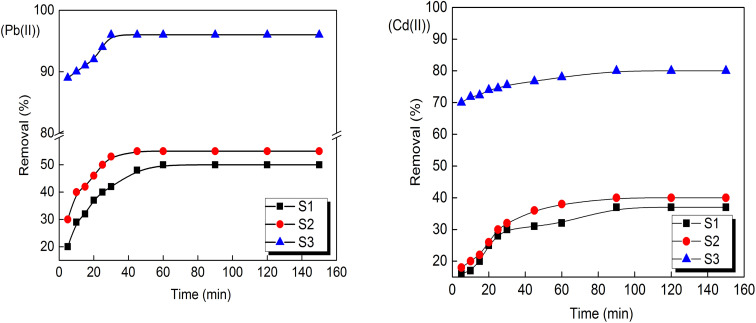
Pb(ii) and Cd(ii) ions removal as a function of contact time by the prepared activated carbon (concentration of adsorbent: 2 g L^−1^; agitation speed: 160 rpm, initial metal concentration under the test: 10 mg L^−1^).

The influence of contact time towards Pb(ii) and Cd(ii) removal by the different prepared porous carbons is displayed in Fig. 3; keeping the Pb(ii) and Cd(ii) metal ions concentration constant 10 mg L^−1^. The removal percentage of both nominated metal ions increased with the increasing contact time until the equilibrium after 30, 60 and 60 min, respectively for the prepared porous carbons S3, S1 and S2 for lead ions adsorption. In the other hand after 90 min equilibrium was reached applying porous carbon S3 for cadmium ions adsorption. Therefore, the obtained porous carbon enclosed with graphene-like layers (S3) that prepared through hydrothermal carbonization of HWC followed by KOH activation poses the highest removal percentage at all tested concentrations. It is found that the removal percentages for Pb(ii) and Cd(ii) onto S3 were 96 and 80%, respectively, at metal solutions of 10 mg L^−1^ concentration. This finding shows that the prepared S3 porous carbon is an effective adsorbent in adsorption of both metal ions.

For S3, the fast adsorption of metal ions at the initial stage can be attributed to it has largest surface area and accessible pores which provides large number of available sites as well as the presence of basic surface groups for adsorption as shown in Fig. 3. By increasing the adsorption time, the occupation by metal ions on the adsorbent surface is reduced due to the saturation of the prepared carbon surface. At equilibrium, a small mass transfer occurs for Pb(ii) or Cd(ii) ions from the bulk liquid to the external surface of adsorbent. The adsorption capacity and removal percentage increased rapidly with decreasing metal ions concentrations, where, at lower concentrations of metal ions (5 mg L^−1^), the removal percentages of S3 for Pb(ii) and Cd(ii) were 97 and 90%, respectively (not shown here in Fig. 3). The prepared S3 still has the largest removal percentage comparing with other adsorbents S1 and S2. Although the ionic radii (Pauling) of the Pb^2+^ (1.20 Å) is larger than that of Cd^2+^ (0.97 Å),^32,33^ it has been noted that the selectivity sequence of metal ions adsorption is Pb > Cd. The reason for greater affinity toward Pb(ii) is attributed to the higher electronegativity of Pb than Cd and also the smaller hydrated radius of Pb(ii) ions in solution (Pb(ii) = 0.401 nm, Cd(ii) = 0.426 nm).^33^ Based on all above results, it can be included that KOH activation of hydrothermally treated HWC produces highly porous graphene-like carbon with basic surface nature and having large total surface area gave rise to effective removal of both metal ions.

**Fig. 4:**
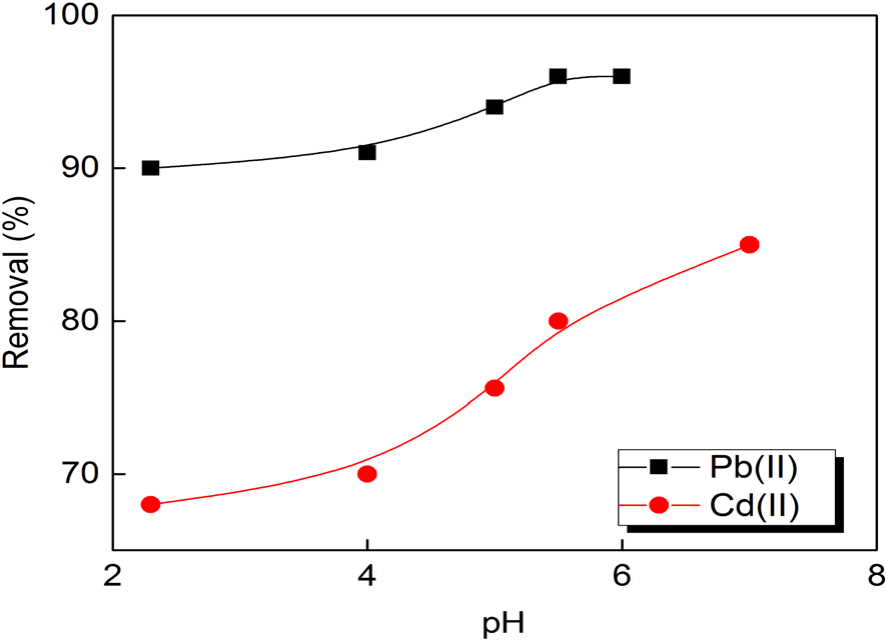
Effect of initial pH value on Pb(ii) and Cd(ii) ions removal by the prepared activated carbon S3 (concentration of adsorbent: 2 g L^−1^; agitation speed: 160 rpm, initial metal concentration: 10 mg L^−1^).

The pH impact on the adsorption process was studied between 2 and 7 and the data reached depicted in Fig. 4 for the prepared S3 sample at optimum time (60 min). With the increase of pH value, the removal percentage for Pb(ii) or Cd(ii) on the prepared porous carbons is increased significantly up to pH 5.5 and 7 for Pb(ii) and Cd(ii) ions, respectively. Accordingly, the experiments were not conducted at pH values higher than 6 to avoid the precipitation of lead ions as hydroxides. For pH values between 3.0 and 4.0, Pb(ii) adsorption removal is changed significantly and the maximum removal attained at pH values 5.5 and no change occurs at pH of 6. The escalation in removal percentage of Cd(ii) attained at pH value of 7. At pH below 4, the excess H^+^ ions form (H_3_O^+^) ions and compete with Pb(ii) and Cd(ii) ions for the surface of the adsorbent. This could be due to the excess of H^+^ ions surrounding the binding sites on the adsorbent surface making the adsorption unfavorable. Increasing pH to 4, there is low competition between the metal ions and for the active sites which lead to the increase in the removal percentages due to the change the state of hydration of heavy metal ions in solution. Similar behaviour was obtained by Acharya *et al.*^34^ and Boudrahem *et al.*^35^ for Pb(ii) removal by activated carbon derived from tamarind wood-derived and coffee residue derived, respectively.

**Fig. 5:**
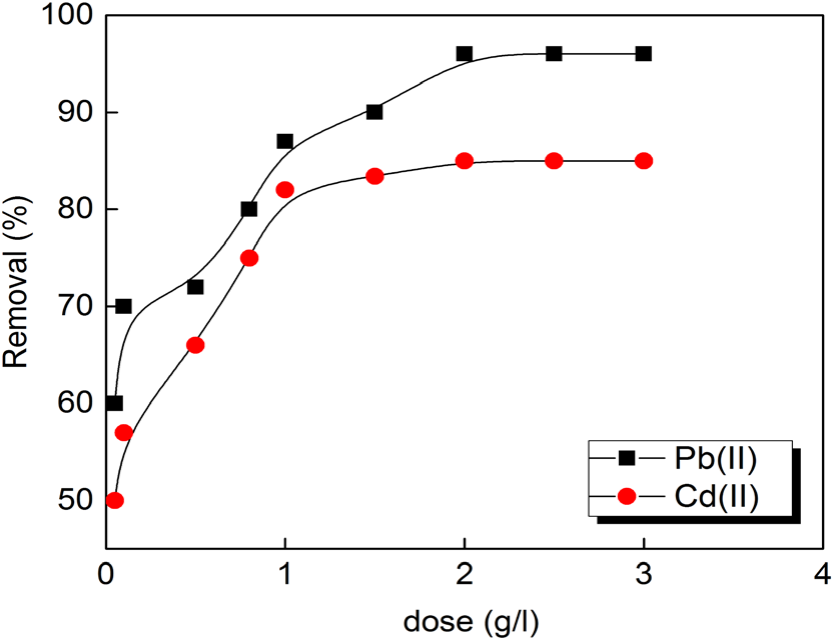
Effect of the prepared activated carbon S3 dose (agitation speed: 160 rpm, initial metals concentrations: 10 mg L^−1^, dose: 2 g L^−1^, pH 5.5 and 7 for Pb and Cd(ii)).

The influence of porous carbon adsorbent's dosage for metal ions adsorption by the prepared composite S3 is displayed in Fig. 5. The removal percentage is increased and attained its optimum value at a dosage of 2 g L^−1^ for the two tested metal ions. The percentage did not vary with increasing the dose of the adsorbent to 3 g L^−1^ and that can be due to the fully occupation of the adsorbents surface active sites and their aggregation. The same results were observed by Momčilović *et al.*^36^ using pine cone activated carbon for Pb(ii) ions removal.

### 3.3. Adsorption studies

Due to their toxic effects, the removal of Pb and Cd(ii) from their aqueous solutions using different adsorbents gained much interest in literature.^11–13,15,32–35,37–42^ During these studies, different kinetic models such as pseudo-first order (PFO), pseudo-second order (PSO) and intraparticle diffusion have been performed to describe the adsorption mechanism. Moreover, the data of equilibrium adsorption were analyzed using Langmuir, Freundlich, Dubinin–Radushkevich and Redlich–Peterson isotherms to estimate the adsorption behavior of metals ions onto the adsorbents.

#### 3.3.1. Adsorption kinetic study

By applying the different kinetic models for the experimental data as shown in Table 2, the PSO model gives the best fitting to the experimental data for both adsorbed metal ions according to the high values of *R*^2^ value. From these results, the adsorption is can be mainly attributed to chemisorption involving valence forces or ion exchange between the adsorbent and metal ions.^41,42^

Isotherm and kinetics models parameters fit

**Table:** 

Metal	Isotherm models		
	Langmuir isotherm parameters		
	*q*_m_ (mg g^−1^)	*K*_L_ (L mg^−1^)	*R* ^2^
Pb(ii)	207.9	0.02	0.98
Cd(ii)	119.6	0.01	0.99

**Table:** 

	Freundlich isotherm parameters		
	1/*n*	*K*_F_ (L mg^−1^)	*R* ^2^
Pb(ii)	0.66	8.8	0.97
Cd(ii)	0.54	5.8	0.95

**Table:** 

	D–R isotherm parameters		
	*q*_DR_ (mg g^−1^)	*E* (kJ mol^−1^)	*R* ^2^
Pb(ii)	115.7	9.3	0.96
Cd(ii)	82.3	8.9	0.95

**Table:** 

	Redlich–Peterson parameters			
	*K*_R_ (L mg^−1^)	*a*_R_ (L mg^−1^)	*g* (g)	*R* ^2^
Pb(ii)	6.8	0.17	0.63	0.96
Cd(ii)	1.8	0.01	1	0.98

**Table:** 

Metal	Kinetic model			
	Pseudo-first order			
	*q*_e,exp._ (mg g^−1^)	*q*_e,Cal_ (mg g^−1^)	*K*_1_	*R* ^2^
Pb(ii)	4.8	4	0.05	0.07
Cd(ii)	4.2	3.5	0.03	0.07

**Table:** 

	Pseudo-second order		
	*q*_e,Cal_ (mg g^−1^)	*K*_2_ (g mg^−1^ min^−1^)	*R* ^2^
Pb(ii)	4.6	0.14	0.99
Cd(ii)	3.8	0.04	0.99

**Table:** 

	Intra-particle diffusion		
	*k*_p_ (mg g^−1^ min^−1/2^)	*C*	*R* ^2^
Pb(ii)	0.03	4.4	0.95
Cd(ii)	0.07	3.4	0.93

**Fig. 6:**
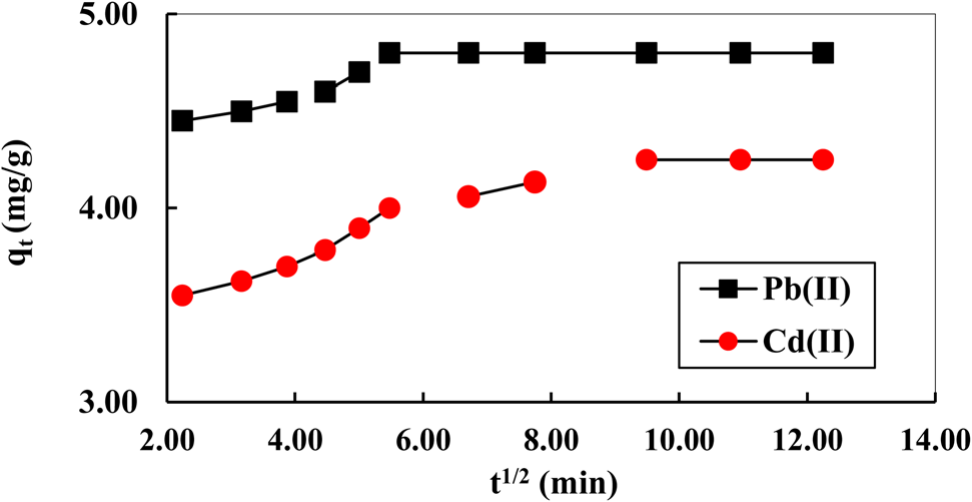
Kinetic stages in intraparticle diffusion plot of Pb(ii) and Cd(ii) ions adsorption on activated carbon S3 (agitation speed: 160 rpm, initial metals concentrations: 10 mg L^−1^, dose: 2 g L^−1^, pH 5.5 and 7 for Pb and Cd(ii)).

The intraparticle diffusion model was used for fitting the experimental data to identify the rate determining step controlling the adsorption process. Generally, the adsorption process includes three steps, namely, the diffusion of adsorbate to the film surrounding (film diffusion) of the adsorbent; diffusion to the internal sites from the film (intra-particle diffusion); and metal ions uptake which can occurs by different mechanisms (*e.g.*, adsorption and complexation).^42^ It is observed from Fig. 6 that multi-linear stages exist in the model plot indicating that the adsorption of metal ions occurred *via* three stages including: external film diffusion in the first step and here the adsorption is due to the diffusion of the ions from the solution to the adsorbent surface through the boundary layer. The final stage is the process accompanied with the equilibrium has the lowest diffusion rate amongst the three stages. As shown in the Fig. 6, the curve did not pass through the origin, revealing that the chemical adsorption governed the adsorption process and thus the adsorption is not only controlled by the intra-particle diffusion.

#### 3.3.2. Adsorption isotherm study

**Fig. 7:**
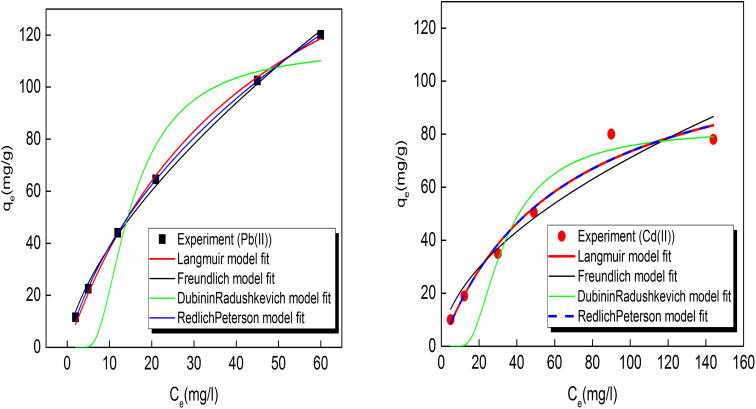
Adsorption isotherms of Pb(ii) and Cd(ii) ions (agitation speed: 160 rpm, initial metals concentrations: 25–300 mg L^−1^, dose: 2 g L^−1^, pH 5.5 and 7 for Pb and Cd(ii)).

The experimental data of Pb(ii) and Cd(ii) ions adsorption by the prepared porous carbon S3 were fitted at various initial concentrations with adsorption isotherm models (Fig. 7) and the obtained parameters values are summarized in Table 2. From the above experimental results, the optimum contact times obtained for Pb(ii) and Cd(ii) ions were 30 and 90 min respectively. Equilibrium attained using a dose of 2 g L^−1^ for both ions at pH values of 5.5 and 7 for Pb(ii) and Cd(ii).

According to the *R*^2^ values obtained from the models fitting, the Pb(ii) and Cd(ii) adsorption on the prepared porous graphene-like carbon (S3) described better with Langmuir isotherm model. The adsorption is decreased at higher concentrations of Pb(ii) and Cd(ii) ions. The fitting results revealed that the of Pb(ii) and Cd(ii) ions adsorption occurs on the homogeneous surfaces of adsorbents by a single layer of chemisorption.^37^ The maximum adsorption capacities (*q*_m_) of porous graphene-like carbon (S3) for Pb(ii) and Cd(ii) ions were 207.9 and 119.6 mg g^−1^, respectively.

Table 3 lists the maximum adsorption capacities (*q*_m_, mg g^−1^) of Pb and Cd(ii) ions over the obtained samples compared with other adsorbents.^13,38–42^ The obtained results of *q*_m_ for Pb and Cd(ii) ions are comparable to results in literature for other adsorbents as shown in Table 3. The adsorption both metal ions by porous carbon S3 activated by KOH can take place by both adsorption through the accessible pores and ion exchange between metal ions attached to the adsorbent after displacing the proton (H^+^) and forming an adsorbent-metal complex. The data obtained are quite fitting for Langmuir isotherm model.

**Table:** Comparison for maximum adsorption capacities (*q*_max_) of different porous carbons toward Pb(ii) and Cd(ii) ions

Adsorbent	*q*_max_ (mg g^−1^)	Reference
Pistachio-wood-derived activated carbon	190.2 Pb(ii)	13
	(pH 6.0 and dosage 0.1 g L^−1^)	
Commercial-activated carbon	23.4 Pb(ii)	38
	(pH 5, initial concentrations <50 to <600 mg L^−1^)	
Water-hyacinth-derived activated carbon	118.8 Pb(ii)	39
	(pH 6.0, contact time 20 min and dosage 1.2 g L^−1^)	
Vatica rassak wood waste-derived activated carbon	149.2 Pb(ii)	40
	(pH 6.0 and dosage 0.1 g L^−1^)	
Activated alumina	35.06 Cd(ii)	41
	(pH 5, dosage 7.5 g L^−1^, contact time 2 h)	
Prepared activated carbon (S3)	207.9 Pb(ii)	This work
	119.6 Cd(ii)	
	(pH 5.5 and 7 for Pb(ii) and Cd(ii) initial concentrations 25–300 mg L^−1^)	

It has been estimated that the adsorption results did follow the Freundlich isotherm model for Cd(ii) adsorption. Freundlich model is known to describe the physical adsorption process and distribution of adsorption sites on energies-heterogeneous surface. When values of Freundlich constant “1/*n*” that relates to the intensity of adsorption equal unity, suggests that the adsorption is independent of concentration. For values of 1/*n* > 1, the process of adsorption is normal, while the value of 1/*n* < 1, the adsorption process is a favorable. The obtained values of 1/*n* were 0.66 for Pb(ii) and 0.54 for Cd(ii) as shown in Table 2, indicating more favorability of the Pb(ii) Cd(ii) ions adsorption on the prepared adsorbent.

According to Polanyi's theory, the D–R model is developed and assumes that the Gaussian energy distribution control the distribution of pores in adsorbent. The values of *q*_KD_ and the free energy (*E*) were calculated from the non-linearized equation and listed in Table 2. It is stated that the adsorption is dominated by physical process when *E* values < 8 kJ mol^−1^, while it occurs by chemical process at 8 < *E* < 16 kJ mol^−1^. The calculated values of *E* were 9.3 and 8.9 kJ mol^−1^ for Pb(ii) and Cd(ii) respectively, which confirm the chemical adsorption process.

On the other hand, R–P model is a hybrid model of both Langmuir and Freundlich models and applied for homogeneous or heterogeneous adsorption processes and when *g* equals to 1, R–P model reduces to the Langmuir model. As shown in Table 2, the values of *g* gives unity for Cd(ii)and close to unity for Pb(ii) ions revealing that Langmuir model is the best model that represents Pb(ii) and Cd(ii) adsorption by porous carbon S3.

### 3.4. Adsorption thermodynamics

**Fig. 8:**
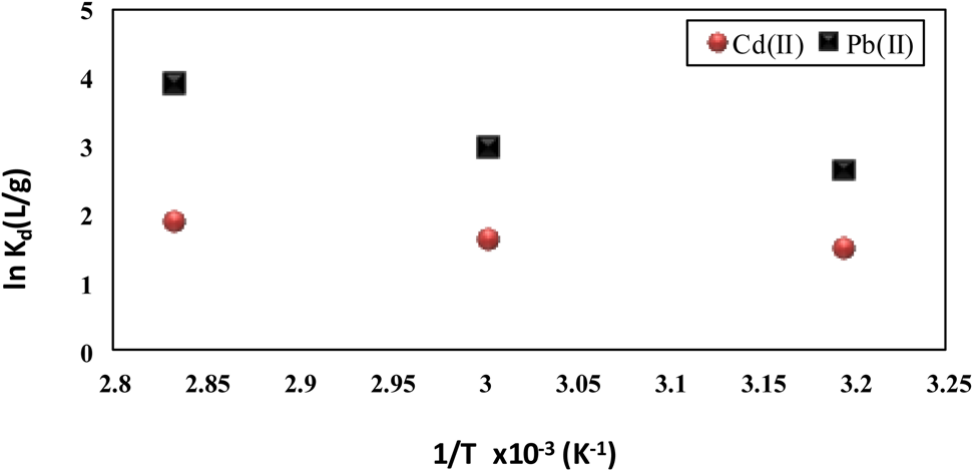
Thermodynamic plot for Pb(ii) and Cd(ii) ions adsorption onto activated carbon S3 (agitation speed: 160 rpm, initial metals concentrations: 10 mg L^−1^, dose: 2 g L^−1^, pH 5.5 and 7 for Pb and Cd(ii)).

The adsorption capacity of Pb(ii) and Cd(ii) on the prepared activated carbon S3 increased with the reaction temperature rising the thermodynamic data reached from applying eqn (12) are represented in Table 4 and Fig. 8. The obtained Δ*G*^0^ values were all of negative values, indicating that the adsorption of two metal ions was spontaneous. The positive values of Δ*H*^0^ indicate that this adsorption is an endothermic process and the adsorption is favorable at high temperatures. Similarly, the values of entropy change (Δ*S*^0^) were positive for both metal ions revealing that the degree of disorder at the solid–solution interface during the adsorption process. Similar results were reported by Huang *et al.*^39^

**Table:** Adsorption thermodynamic parameters for Pb(ii) and Cd(ii) ions by porous carbon S3

Metal ions	Δ*G*^0^ (kJ mol^−1^)			Δ*H*^0^ (kJ mol^−1^)	Δ*S*^0^ (J mol^−1^ K^−1^)
	313 K	333 K	353 K		
Pb(ii)	−6.8	−8.2	−11.4	29	113.7
Cd(ii)	−3.9	−4.4	−5.4	8.2	38.4

### 3.5. Reusability and regeneration

**Fig. 9:**
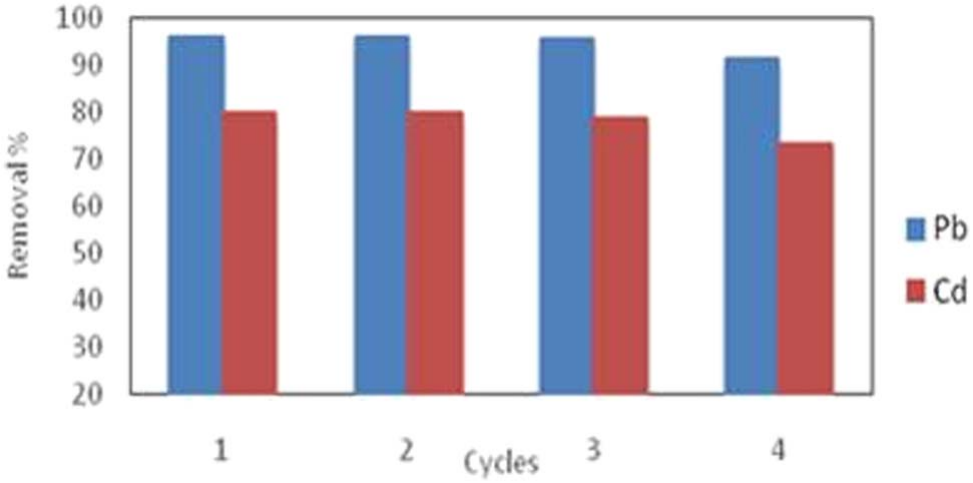
Reusability efficiency of activated carbon S3 for desorption of Pb(ii) and Cd(ii) ions (agitation speed: 160 rpm, initial metals concentrations: 10 mg L^−1^, dose: 2 g L^−1^, pH 5.5 and 7 for Pb and Cd(ii)).

The excellent recycling performance of the adsorbent determines its ability for application in the environment. The adsorption capacity of adsorbent is almost maintained in the first cycle and then the removal started to be slightly reduced. Results in Fig. 9 indicated that the successive regeneration cycles for S3 resulted in the reduction of the desorption efficiency by 5% and 8% for Pb(ii) and Cd(ii) ions, respectively; after the fourth cycles. Therefore, such results revealed that S3 could be recycled with high desorption performance which is relating to its porous graphene-like structure and Lewis base functional groups throughout the successive four desorption cycles. Similar results were reported by others.^39,40^

## 4. Conclusion

Porous carbon enclosed with graphene was successfully prepared by hydrothermal carbonization at 750 °C and alkaline activation for hard wood composite waste as adsorbent precursor. The synthesized sample displayed good activity for Pb(ii) and Cd(ii) ions removal. FE-SEM and TEM investigations confirmed the formation of graphene-like carbon samples (S2 and S3). KOH activating agent generated micro-mesoporous structure with the highest total surface area in S3. FTIR results revealed that C–O and OH groups might be involved in Pb(ii)/Cd(ii) adsorption onto S3. The interaction of Pb(ii) and Cd(ii) and the functional groups present on carbon surface could also occur by physical adsorption (*i.e.*, electrostatic attraction forces). The adsorption capacity of Pb(ii) was higher than that of Cd(ii) and the best results obtained by the prepared sample S3 based on the higher electronegativity and smaller hydrated radius of Pb(ii) ions. The kinetic modeling for the experimental data indicated that pseudo second order described the adsorption of metal ions optimal. The Langmuir isotherm model is the better isotherm described metal ions adsorption with maximum adsorption removal of 207.9 and 119.6 mg g^−1^ for Pb(ii) and Cd(ii), respectively; under optimal conditions of carbon dose (2 g L^−1^) and pH 5.5 and 7 for Pb and Cd(ii) and room temperature (25 °C). Such result may be attributed to presence of Lewis base groups and high surface area accompanied by enclosing graphene sheets through hydrothermal carbonization. Moreover, the uptake of Pb and Cd(ii) ions may be followed by physico-chemical surface adsorption. Metal ions adsorption by S3 was a spontaneous process and endothermic reaction was concluded from the calculated thermodynamic data. Therefore, the obtained porous carbons from hard wood composite waste can candidate as an economic and effective adsorbent for uptaking heavy metals polluted water. In conclusion, the studied strategy can be considered as a good means for recycling HWC waste at the end of their service period to produce added-value and smart materials such as porous graphene-like carbon.

## Conflicts of interest

The authors declare no conflict of interest.

## Supplementary Material
